# Oestrogen receptors interact with the α-catalytic subunit of AMP-activated protein kinase

**DOI:** 10.1042/BSR20150074

**Published:** 2015-10-30

**Authors:** Yulia Lipovka, Hao Chen, Josef Vagner, Theodore J. Price, Tsu-Shuen Tsao, John P. Konhilas

**Affiliations:** * Department of Molecular and Cellular Biology, University of Arizona, Tucson, AZ 85724, U.S.A.; † Department of Physiology, University of Arizona, Tucson, AZ 85724, U.S.A.; ‡ Sarver Molecular Cardiovascular Research Program, University of Arizona, Tucson, AZ 85724, U.S.A.; § The BIO5 Research Institute, University of Arizona, Tucson, AZ 85724, U.S.A.; ║ Department of Pharmacology, University of Arizona, Tucson, AZ 85724, U.S.A.; ¶ Department of Biochemistry and Molecular Biophysics, University of Arizona, Tucson, AZ 85724, U.S.A.

**Keywords:** AMP-activated protein kinase (AMPK), breast cancer, oestradiol, oestrogen receptors

## Abstract

Normal and pathological stressors engage the AMP-activated protein kinase (AMPK) signalling axis to protect the cell from energetic pressures. Sex steroid hormones also play a critical role in energy metabolism and significantly modify pathological progression of cardiac disease, diabetes/obesity and cancer. AMPK is targeted by 17β-oestradiol (E2), the main circulating oestrogen, but the mechanism by which E2 activates AMPK is currently unknown. Using an oestrogen receptor α/β (ERα/β) positive (T47D) breast cancer cell line, we validated E2-dependent activation of AMPK that was mediated through ERα (not ERβ) by using three experimental strategies. A series of co-immunoprecipitation experiments showed that both ERs associated with AMPK in cancer and striated (skeletal and cardiac) muscle cells. We further demonstrated direct binding of ERs to the α-catalytic subunit of AMPK within the βγ-subunit-binding domain. Finally, both ERs interacted with the upstream liver kinase B 1 (LKB1) kinase complex, which is required for E2-dependent activation of AMPK. We conclude that E2 activates AMPK through ERα by direct interaction with the βγ-binding domain of AMPKα.

## Abstract (precis)

We identified a novel interaction between the classical oestrogen receptors (ERα and ERβ) and the catalytic subunit of AMP-activated protein kinase (AMPK) in several cell types. In addition, we demonstrate that oestradiol (E2) activates AMPK through ERα and requires the upstream kinase complex liver kinase B (LKB1).

## INTRODUCTION

As a central metabolic sensor that helps maintain cellular energy homoeostasis, AMP-activated protein kinase (AMPK) has emerged as a key player in many metabolic processes including glucose up-take and fatty acid oxidation in striated muscle, fatty acid synthesis and gluconeogenesis in the liver and the regulation of food intake in the hypothalamus [1,2]. AMPK is sensitive to any condition that depletes cellular ATP placing AMPK at the centre of physiological [3] and pathological processes including cancer [4] and cardiovascular disease [5].

AMPK is a heterotrimeric enzyme complex consisting of a catalytic α-subunit and regulatory β- and γ-subunits. Increased intracellular AMP allosterically activates AMPK and permits phosphorylation of the α-catalytic subunit at Thr^172^ [6–8]. This triggers a global metabolic response to energy deprivation leading to up-regulation of ATP producing pathways and down-regulation of energy consuming processes [7]. So far, two kinases that phosphorylate AMPK have been identified: the upstream liver kinase B 1 (LKB1) complex and calcium–calmodulin-dependent protein kinase kinase β (CaMKKβ) [9–11]. The LKB1 complex consists of LKB1 and two accessory subunits, Ste20-related adaptor (STRAD) and mouse protein 25 (MO25), both of which are required for LKB1 activity [10,12].

LKB1 was first identified as a tumour suppressor gene inactivated in patients with Peutz–Jegher's syndrome [13] and, more recently, in certain types of breast cancer [14]. Moreover, AMPK activity is linked to stress resistance and survival in tumour cells [15,16] and inactivation of AMPK accelerates tumorigenesis [17]. Not only is the LKB1/AMPK signalling axis an integral component of the tumour suppressor network, it is also a critical regulator of energy homoeostasis in the heart under normal conditions and during cardiac disease progression [18,19]. In the heart, AMPK is activated during periods of cardiac ischaemia [5]. Yet, long-term activation of AMPK in the heart leads to glycogen accumulation and overt cardiomyopathy [20]. The findings that AMPK can also promote or antagonize cancer [4], similar to cardiac disease, demonstrate that AMPK is central to the energetic balance and can dictate the course of disease progression in multiple cell systems.

It is likely that appropriate manipulation of AMPK activity in patients with certain types of cancer or cardiovascular disease may have a therapeutic benefit. However, adding to the complexity of developing AMPK-based therapies is the fact that both cardiovascular- and cancer-based diseases have significant sex dimorphisms. Although genetic and environmental factors contribute to these sex differences [21], the sex hormone, oestrogen, plays a central role. 17-β-oestradiol (E2) is one of the three major naturally occurring hormones in women whose effects are mediated by the intracellular oestrogen receptors (ER) and by a membrane oestrogen-binding element [22]. E2-induced changes are primarily mediated through the two classical ERs (ERα and ERβ) found in the nucleus and discrete cellular pools in the cytoplasm, which after binding to E2 orchestrate genomic (transcriptional) and non-genomic functions.

Several studies have shown that E2 activates AMPK by increasing the phosphorylation of its α-catalytic subunit. However, the mechanism that leads to E2-induced increase in AMPK phosphorylation remains unknown [23–26]. We wished to further elucidate the impact of E2 on AMPK activity by exploring the specific roles of ERs (ERα and ERβ) during the process of E2-induced AMPK activation. We hypothesized that E2 activates AMPK through direct interaction of ERα and ERβ with the AMPK–LKB1 complex to potentiate post-translational activation of AMPK and AMPK targets. To do this, we exploit an ERα and ERβ positive breast cancer cell line (T47D) to show that E2-dependent activation of AMPK is mediated through ERs, specifically, ERα. We further employ a co-immunoprecipitation strategy not only to demonstrate the ability of the ERs to interact with AMPK in breast cancer and cardiac cells, but also to define the specific region of interaction.

## MATERIALS AND METHODS

### Cells and reagents

293-T and C2C12 cells were purchased from the A.T.C.C. T47D and MDA-MB-231 cells were gifts from Dr Joyce Schroeder from University of Arizona Cancer Center. Neonatal rat cardiomyocytes (NRCM) were kindly provided by Dr Carol Gregorio (University of Arizona). pcDNA–eGFP–ERα and pcDNA–flag–ERβ were purchased from Addgene, pcDNA–myc–GFP, pcDNA–myc–AMPKα2 and myc-tagged AMPKα deletion constructs were gifts from Dr Tsu-Shuen Tsao, University of Arizona. Phospho-AMPKα Thr^172^, AMPKα, phospho-ACC (acetyl-CoA carboxylase), ACC, flag, myc, MO25 and LKB1 antibodies were from Cell Signaling Technology. ERα and ERβ antibodies were from Santa Cruz Biotechnology. eGFP antibody was from Origene. MO25 siRNA, as well as Lipofectamine 2000 were purchased from Invitrogen. 17-β-E2 and actinomycin D were purchased from Sigma–Aldrich. Diarylpropionitrile (DPN) was from Santa Cruz Biotechnology. PHTPP (4-[2-Phenyl-5,7-*bis*(trifluoromethyl)pyrazolo[1,5-*a*]pyrimidin-3-yl]phenol) (TPP) and PPT (4,4′,4″-(4-Propyl-[1*H*]-pyrazole-1,3,5-triyl)*tris*phenol) were from Tocris Bioscience. G-15 ((3aS*,4R*,9bR*)-4-(6-Bromo-1,3-benzodioxol-5-yl)-3a,4,5,9b-3*H*-cyclopenta[*c*]quinoline) was from Azano Pharmaceuticals. The Co-Immunoprecipitation Kit and BCA protein Assay Kit were purchased from Thermo Scientific.

### Preparation of AMPK activator

An AMPK activator, *N*-{4-[3-(1-methyl-cyclohexylmethyl)-2,4-dioxothiazolidine-5-ylidene-methyl]-phenyl]-4-nitro-3-trifluoromethyl-benzenesulfonamide (compound OSU-53), was prepared with small modifications of previously published procedure [27]. The crude compound was purified by column chromatography using silicagel 60 μm and HPLC. Preparative HPLC was performed on a 19×256 mm Waters X-Bridge Preparative C18 column. The mobile phase was 10%–90% acetonitrile and water containing 0.1% trifluoroacetic acid (TFA) within 50 min. The flow rate was 10 ml/min. The dual UV detector system operated at 230 and 280 nm. ESI experiments were performed on an ESI Bruker Apex Qh 9.4 T FT-ICR instrument using standard ESI conditions. Compound was 97%+ pure from analytical HPLC. Analytical HPLC (3.0×150 mm 3.5 μm Waters C18 X-Bridge column, flow rate 0.3 ml/min, linear gradient from 10%–90% B in A over 45 min, where A is 0.1% TFA in water and B is 0.1% TFA in acetonitrile, detection at 220 and 280 nm). NMR spectra were recorded at 300 MHz for ^1^H-NMR and at 125 MHz for ^13^C-NMR.

### Cell culture, transfection and treatments

T47D and MD-MBA-231 cells were maintained in tissue culture dishes (10 cm) in RPMI (Roswell Park Memorial Institute medium) medium (Cellgro) containing 10% FBS, 100 units/ml penicillin and 100 μg/ml streptomycin at 37°C in a humidified incubator supplied with 5% CO_2_. siRNA transfections were performed using 100 pmoles siRNA and Lipofectamine 2000 according to Invitrogen protocol. Briefly, when cells reached 40% confluence 100 pmoles siRNA in 250 μl OPTI-MEM I (improved Minimal Essential Medium) (Gibco, Life Technologies) was mixed with 5 μl of Lipofectamine in 250 μl of OPTI-MEM I and added to the cells in six-well plates along with 500 μl of fresh OPTI-MEM I to a final volume of 1 ml. Four to six hours after transfection the media was changed to fresh 10% FBS RPMI.

For chemical treatments, cells were cultured in six-well plates to 60%–70% confluence and then incubated in serum/phenol red-free RPMI medium for 24 h. For the receptor antagonist experiments, the specific amounts of TPP or G15 were added to the wells for 45 min. Immediately after that and without changing the cell culture media, E2 was added to the wells and incubated for an additional 1 h. Afterwards, the cells were lysed and proteins were extracted.

293-T, C2C12 and NRCM cells were maintained in tissue culture dishes (10 cm) in Dulbecco's modified Eagle's medium (DMEM) (Gibco, Life Technologies) containing 4.5 g/l glucose and supplemented with 10% FBS, 100 units/ml penicillin, 100 μg/ml streptomycin and 2 mM L-glutamine at 37°C in a humidified incubator supplied with 5% CO_2_. Cells were transfected with 5μg of pcDNA constructs using the calcium phosphate precipitation method.

### Co-immunoprecipitation assay

T47D cells were incubated in serum/phenol red-free RPMI medium 24 h before treatment. Cells were left untreated or treated with E2 (10 μM) for 1 h followed by cell lysis. ERs were immunoprecipitated using anti-ERα and anti-ERβ antibody coated columns. 293-T cells were lysed 48 h after transfection and immunoprecipitated using anti-eGFP and anti-flag antibody coated columns.

### Western blot

Cell lysates were analysed for protein concentration using BCA protein assay and then loaded in equal amounts to a SDS/PAGE gel for separation, followed by transfer to a PVDF membrane. Specific antibodies were used to identify the proteins of interest. All immunoblot analyses were performed from the semi-quantification of individual blots and were not compared across blots according to accepted guidelines. To determine the pAMPKα_T172_–AMPKα or pACC–ACC ratios, pAMPKα_T172_ or pACC were determined in each sample. Phospho-proteins were then normalized to total AMPKα or ACC in matched samples. Some immunoblot images of a given target were cropped from the same blot in order to conserve figure space and redundancy.

### AMPK activity assay

AMPK activity of cultured cells was measured using Cyclex AMPK Kinase Assay Kit (Cyclex, MBL International). Briefly, 50 μl of cell lysate was added to and incubated in wells pre-coated with a substrate–peptide corresponding to the amino acid sequence surrounding mouse insulin receptor substrate 1 (IRS-1) Ser^789^, which contains the serine residue that can be efficiently phosphorylated by AMPK. An anti-phospho-mouse IRS-1 Ser^789^ monoclonal antibody was used to detect the phosphorylation of the substrate–peptide.

### Real-time PCR

Total RNA was isolated from the cells using the RNeasy Mini Kit (Qiagen) according to the manufacturer's protocol. cDNA was generated using the Maxima First Strand cDNA Synthesis Kit for RT-qPCR (Thermo Scientific). Maxima SYBR Green qPCR Master Mix (Thermo Scientific) was used for real time PCR reactions. 18S RNA was used as an internal control for real time PCR.

### Statistical analysis

Each experimental series was performed at the same time and was then repeated at least three times on separate days. Then, results are presented as mean values of the experimental groups. A paired *t*test (where appropriate) or a one-way ANOVA with Tukey's post-test was used to compare differences between mean values. Experimental groups in bar graph representations are normalized to control groups and displayed as relative activation. *P*-values of <0.05 were considered statistically significant.

## RESULTS

### Oestrogen receptor α mediates E2-dependent activation of AMPK

Both *in vivo* (using skeletal muscle) and *in vitro* (using skeletal, endothelial, liver and vascular cell lines) studies demonstrate that E2 is capable of activating AMPK [23–26]. Considering the complex role of oestrogen signalling in breast cancer [28] and the findings that AMPK can promote or antagonize cancer [4], we set out to determine a mechanistic link between E2, AMPK activity and ERs using T47D cells, a metastatic, mammary gland cell line expressing both ERs [29]. AMPK activity was assessed by western blot analysis on cell lysates targeting total and phosphorylated AMPKα at Thr^172^ (pAMPKα_T172_) and ACC, a well-characterized AMPK cellular substrate [30,31].

To determine the effective dose of E2 under our experimental conditions and to validate E2-dependent activation of AMPK, we treated T47D cells with an increasing amount of E2 for 1 h. Increased AMPK activity, as measured by pAMPKα_T172_, was observed at all E2 concentrations (except 1 μM; Figure 1A). AMPK targeting of ACC (pACC) was only elevated at 1 and 10 μM (Figure 1A). A time course in T47D illustrates that AMPK activation peaked within 15 min similar to previous studies (Supplementary Figure S1A) [23–26]. A similar dose- and time-dependent response to E2 was observed in C2C12 cells (Supplementary Figure S1B), a mouse myoblast cell line known to show E2-dependent AMPK activation [32]. This activation of the AMPK pathway by E2 is similar to previous studies at similar E2 levels [32,33]. Furthermore, levels of pAMPKα_T172_ and pACC following E2 treatment were significantly less than levels following treatment with an AMPK activator, OSU 53 [27]. Although these previous studies suggest maximal activation at 10 μM E2, our data suggest that this amount of E2 (10 μM) did not maximally activate or saturate AMPK activity.

**Figure 1:**
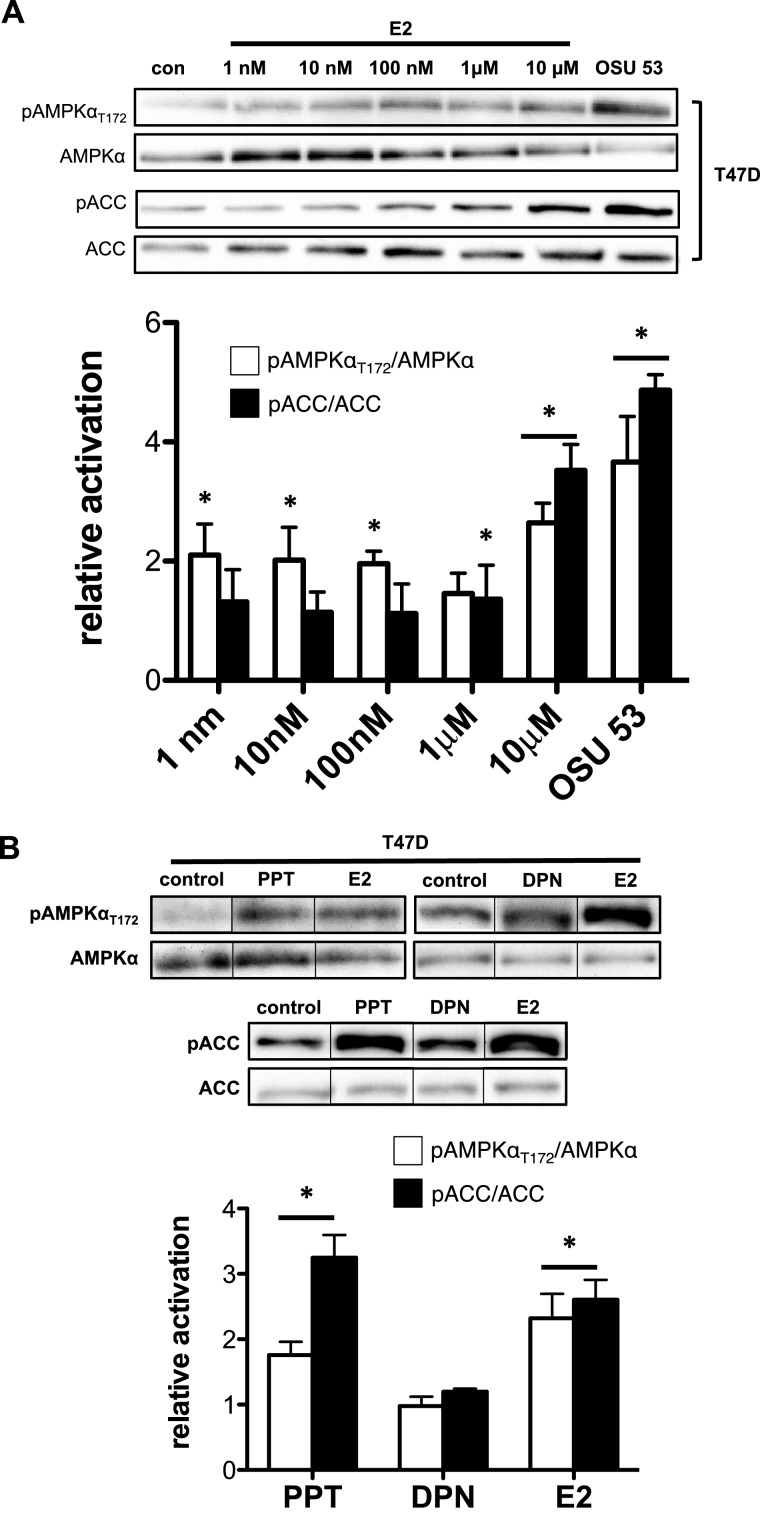
E2 activates AMPK through ERα but not ERβ in T47D cells T47D cells were treated with increasing E2 concentrations (1 nM–10 μM) for 1 h; a western blot analysis was performed on total cell lysate. OSU 53: AMPK activator. (**A**) Western blot (top panel) and bar graph representation (bottom panel) of pAMPKα_T172_–AMPKα ratio and pACC–ACC ratio. T47D cells were treated with 1 μM PPT (ERα agonist), 1 μM DPN (ERβ agonist) or 10 μM E2 for 1 h; a western blot analysis was performed on total cell lysate. (**B**) Western blot (top panel) and bar graph representation (bottom panel) of pAMPKα_T172_–AMPKα ratio and pACC–ACC ratio of total cell lysates from T47D cells treated with indicated compounds. (Treatment groups are normalized to control group to yield relative activation; *P<0.05 from control group.)

ERα and ERβ both regulate distinct ligand-activated transcriptional and non-transcriptional pathways within a cell [34]. Therefore, we determined whether AMPK activation and ACC targeting is mediated through ERα, ERβ or both. To do this, we treated T47D cells with a specific ERα (PPT) or ERβ (DPN) agonist for 1 h. Using pAMPKα_T172_ as an indicator of AMPK activation, we detected an increase in pAMPKα_T172_ with the ERα agonist, PPT but not the ERβ agonist, DPN (Figure 1B). Similarly, western blot analysis of AMPK-target activation paralleled pAMPKα_T172_ showing an elevation in pACC after treatment with the ERα agonist, PPT but not the ERβ agonist, DPN (Figure 1B). These data suggest that AMPK activation and subsequent downstream targeting is an ERα-dependent event. It is important to note that we chose an E2 treatment protocol to match the extent of pAMPKα_T172_ and pACC elevation after treatment with PPT. Because the levels of pAMPKα_T172_ and pACC following E2 or PPT treatment were significantly less than levels following treatment with a pure AMPK agonist, OSU 53 [27] (Figure 1A; Supplementary. Figure S1), the E2 treatment protocol did not maximally activate or saturate AMPK activity.

Next, we used three additional strategies to confirm that E2-dependent activation of AMPK is, indeed, mediated by ERα. First, MDA-MB-231 cells, an ERα negative cell line with ERβ expression and an intact AMPK singling network (Supplementary Figures S2A and S2B) [35,36], were pre-treated for 45 min with an ERβ antagonist (TPP) followed by treatment with E2 with continued exposure to TPP for 1 h. As expected, E2 treatment of MDA-MB-231 cells in the presence or absence of the ERβ antagonist, TPP, did not increase pAMPKα_T172_ or pACC (Figure 2A), whether measured by western blot or AMPK catalytic activity (Figure 2C) [37]. Second, T47D cells were similarly pre-treated with TPP followed by treatment with E2 for 1 h. Treatment with the ERβ antagonist (TPP) did not prevent E2-dependent activation of AMPK. Instead, TPP treatment potentiated the impact of E2 on AMPK activation (pAMPKα_T172_) and target activity (pACC) by 3–5-fold over E2 treatment alone (Figure 2B). Finally, we genetically silenced ERβ expression using ERβ siRNA in T47D cells, which suppressed its expression by 60% (Supplementary Figure S2C). Similar to TPP treatment in T47D cells, E2 treatment in T47D cells following ERβ knockdown did not inhibit E2-dependent AMPK activation but potentiated AMPK activation, when compared with cells with intact ERβ expression levels (Figure 2D).

**Figure 2:**
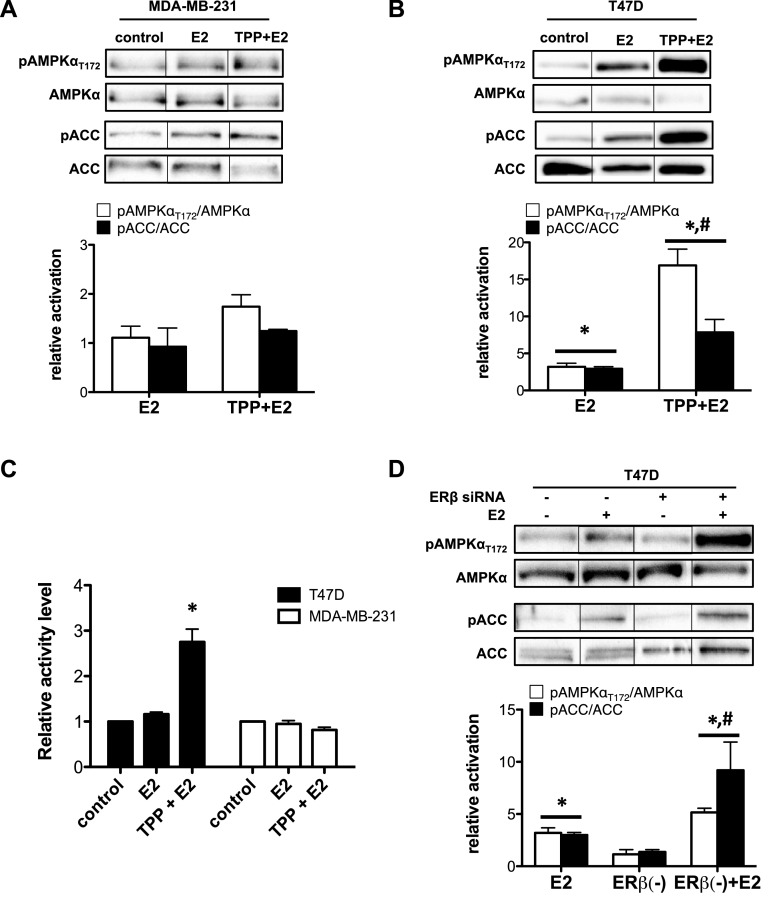
AMPK activation by E2 is dependent on ERα expression and affected by selective ERβ antagonism MDA-MB-231 (ERα deficient) or T47D cells were pre-treated with 10 μM TPP (ERβ antagonist) for 45 min and then treated with 10 μM E2 for 1 h with continued exposure to TPP. Western blot analysis was performed on total cell lysate. (**A**) Western blot (top panel) and bar graph representation (bottom panel) of pAMPKα_T172_–AMPKα ratio and pACC–ACC ratio of total cell lysate from MDA-MB-231 cells. (**B**) Western blot (top panel) and bar graph representation (bottom panel) of pAMPKα_T172_–AMPKα ratio and pACC–ACC ratio of total cell lysate from T47D cells. (Treatment groups are normalized to control group to yield relative activation; *P<0.05 from control group; #*P*<0.05 from E2-treated group). (**C**) AMPK activity assay (ELISA) on total cell lysates from T47D and MDA-MB-231 cells pre-treated with 10 μM TPP (ERβ antagonist) for 45 min and then treated with 10 μM E2 for 1 h. (*P<0.05 from all other groups.) (**D**) Western blot (top panel) and bar graph representation (bottom panel) of pAMPKα_T172_–AMPKα ratio and pACC–ACC ratio of total cell lysate from T47D cells transfected with ERβ siRNA and treated with 10 μM E2 for 1 h. (*P<0.05 from control group; #*P*<0.05 from E2-treated group.)

### Oestrogen receptors α and β directly interact with AMPKα

Acting as ligand-dependent factors, ERα and ERβ bind DNA to control gene activity or interact with other proteins to control signalling cascades [34,38]. Therefore, we further hypothesized that the mechanism of E2-dependent activation of AMPK is through direct interaction of ERs and AMPK subunits. Accordingly, we investigated the interaction of AMPKα with the two ERs (ERα and ERβ) by performing co-immunoprecipitation experiments. Following 1-h treatment with E2 as described above, lysates from T47D cells were added to either ERα or ERβ antibody coated columns. The eluted lysates were analysed by SDS/PAGE followed by western blot analysis for the catalytic AMPKα subunit. Western blot analysis of eluates from ERα or ERβ antibody coated columns showed the presence of AMPKα (Figure 3A). The intensity of association did not appear to change significantly with E2 treatment compared with non-treated cells.

**Figure 3:**
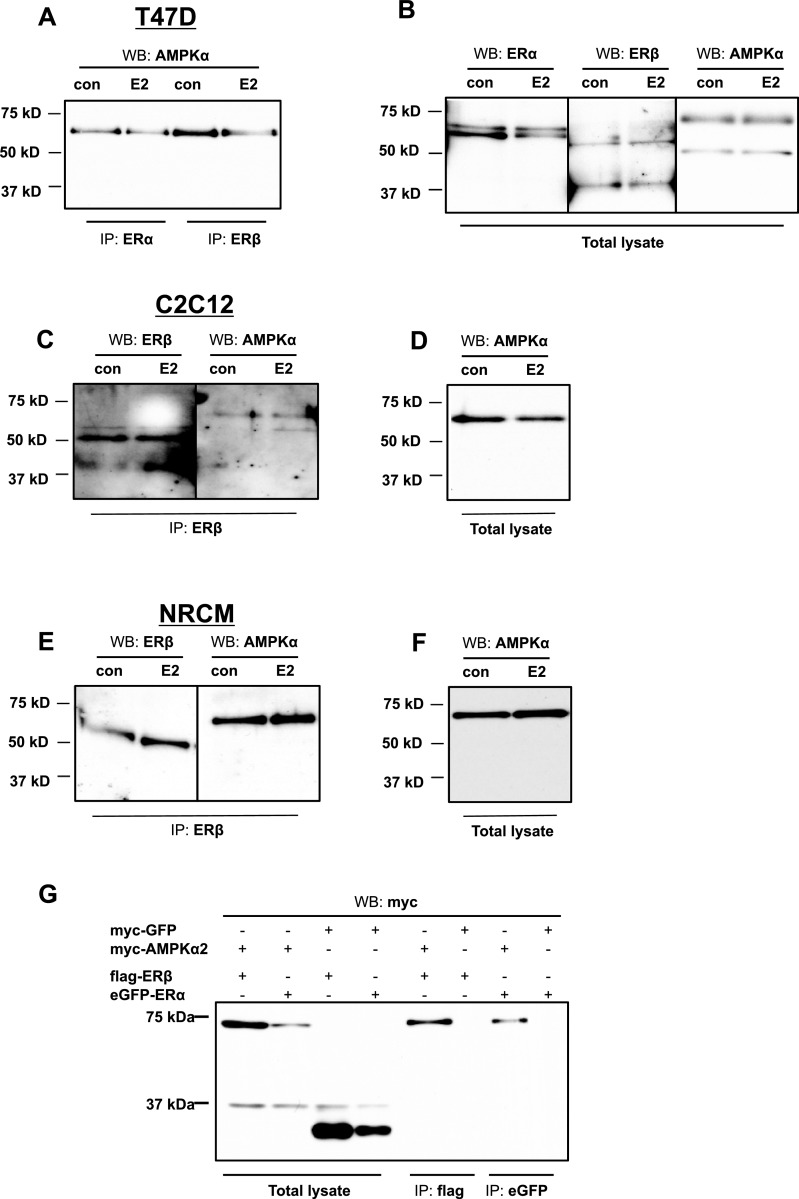
ERα and ERβ interact with the catalytic subunit (AMPKα) of AMPK in T47D cells, C2C12 cells and NRCM (**A**) T47D cells were treated with 10 μM E2 for 1 h. Cell lysates were immunoprecipitated with anti-ERα (IP: ERα) or anti-ERβ (IP: ERβ) antibody, followed by western blot analysis using anti-AMPKα (WB: AMPKα) antibody. (**B**) The same lysates were immunoblotted directly using anti-ERα (WB: ERα), anti-ERβ (WB: ERβ) and anti-AMPKα (WB: AMPKα) antibodies. (**C**) C2C12 cells were treated with 10 μM E2 for 1 h. Cell lysates were immunoprecipitated with anti-ERβ (IP: ERβ) antibody, followed by western blot analysis using anti-AMPKα (WB: AMPKα) antibody. (**D**) The same lysates were immunoblotted directly using anti-AMPKα (WB: AMPKα) antibody. (**E**) NRCM were treated with 10 μM E2 for 1 h. Cell lysates were immunoprecipitated with anti-ERβ (IP: ERβ) antibody, followed by Western blot analysis using anti-AMPKα (WB: AMPKα) antibody. (**F**) The same lysates were immunoblotted directly using anti-AMPKα (WB: AMPKα) antibody. (**G**) 293-T cells were co-transfected with constructs expressing flag-ERβ, eGFP–ERα, myc-AMPKα2 and myc-GFP. Cell lysates were immunoprecipitated with anti-flag (IP: flag) or anti-eGFP (IP: eGFP) antibody, followed by western blot analysis using anti-myc (WB: myc) antibody.

We also wanted to see if this interaction was conserved between different cell systems. Accordingly, we repeated the co-immunoprecipitation experiments in C2C12 cells and NRCM. Once again, following 1-h treatment with E2, lysates from C2C12 cells or NRCM were added to ERβ antibody coated columns and then analysed by western blot for the catalytic AMPKα subunit. We found similar results as in T47D cells, with AMPKα being present in the eluates from C2C12 (Figure 3C) and NRCM (Figure 3E) suggesting association with ERβ.

To determine whether the interaction between ERα/ERβ and AMPKα is a consequence of direct association, we overexpressed eGFP-tagged ERα or flag-tagged ERβ in combination with myc-tagged AMPKα2 (one of two AMPKα isoforms) or myc-tagged GFP (negative control for binding) in 293-T cells. After harvesting the cells, we co-immunoprecipitated cell lysates using anti-flag or anti-eGFP coated columns and performed a western blot on the eluted samples using an antibody that recognizes myc on the myc-tagged AMPKα2. Our results showed that AMPKα2 binds to ERα, as well as ERβ (Figure 3G). We confirmed that the pull-down product was indeed AMPKα2 using LC–MS/MS (Arizona Proteomics Consortium). Pulldown of ERs was confirmed by western blotting (Supplementary. Figure S3).

### Oestrogen receptors α and β directly interact with the βγ-binding domain of AMPKα

AMPKα is composed of a catalytic kinase domain (KD), an auto-inhibitory domain (AID) and a βγ-binding domain [39]. Knowing that the α-subunit of AMPK can directly interact with both ERs, we wanted to determine the specific domain of AMPKα that harbours the binding site specific to ERα or ERβ or both. To do this, we used previously generated myc-tagged AMPKα deletion constructs that contain the catalytic KD (1–312), catalytic kinase and autoinhibitory domains (AIDs) (1–398), auto-inhibitory and βγ-binding domains (303–552) or βγ-binding domain (386–552) of AMPKα2 (Figure 4A). Each of these constructs was co-transfected with eGFP-tagged ERα or flag-tagged ERβ in 293-T cells. Then, we co-immunoprecipitated the cell lysates using anti-eGFP (ERα) or anti-flag (ERβ) antibodies followed by SDS/PAGE and western blotting.

**Figure 4:**
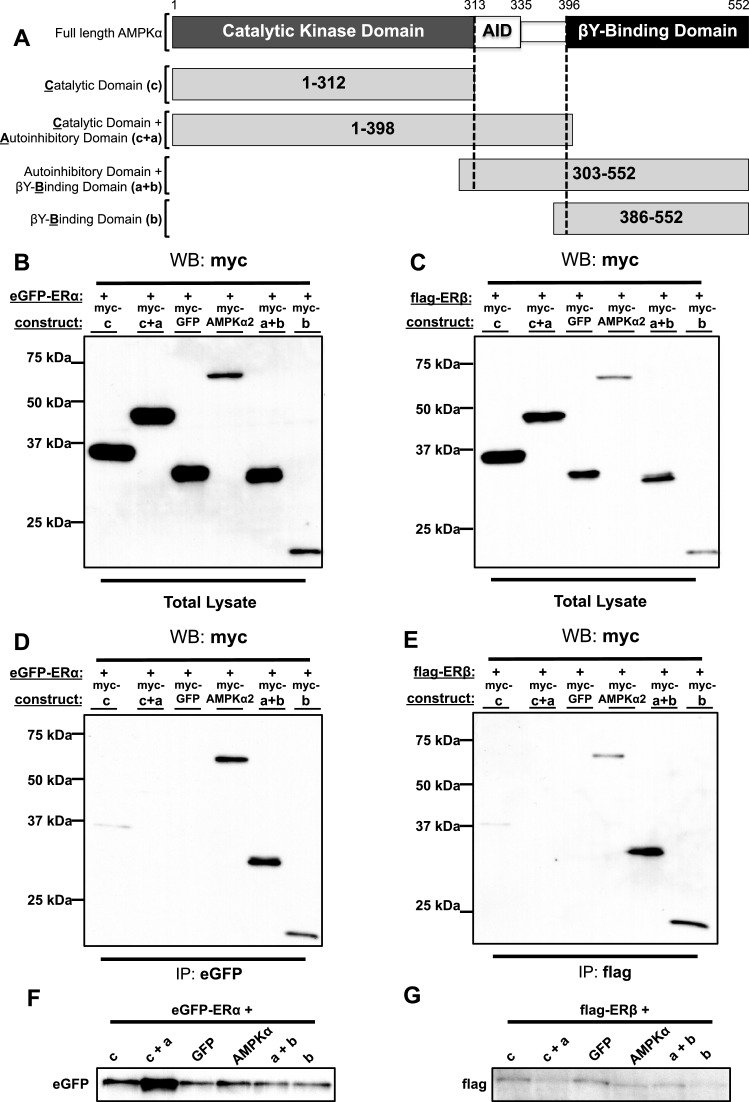
The βγ-binding domain of AMPKα2 binds to the ERs (**A**) A schematic of AMPK illustrating its full-length α-catalytic subunit and the AMPKα deletion constructs. 293-T cells were co-transfected with full-length myc-AMPKα2, myc-tagged AMPKα2 deletion constructs or myc-GFP along with eGFP–ERα or flag–ERβ. Cell lysates were co-immunoprecipitated using anti-eGFP (IP: eGFP) or anti-flag (IP: flag) antibody followed by a western blot analysis using anti-myc (WB: myc) antibody. (**B** and **C**) Western blot analysis of total cell lysates. (**D**) Western blot analysis of eGFP immunoprecipitated cell lysates. (**E**) Western Blot analysis of flag immunoprecipitated cell lysates. (**F**) Western blot analysis using anti-eGFP antibody on cells transfected with eGFP–ERα expression construct. (**G**) Western blot analysis using anti-flag antibody on cells transfected with flag-ERβ expression construct.

First, we confirmed expression of AMPKα2 full length or deletion constructs including the GFP-myc construct as a negative binding control using the anti-myc antibody (Figures 4B and 4C). Similarly, ERα or ERβ were co-expressed with each of the AMPKα constructs (Figures 4F and 4G). As shown in Figure 4(D), we found that ERα bound strongly to constructs a + b (auto-inhibitory and βγ-binding domains) and b (βγ-binding domain) but not constructs c (catalytic KD), c + a (catalytic kinase and AIDs) or GFP-myc (anti-eGFP does not interact with GFP) suggesting interaction with the βγ-binding domain of AMPKα, only. An identical pattern of interaction was observed in cells co-transfected with ERβ and AMPKα constructs (Figure 4E).

### Disruption of the LKB1–MO25–STRAD activation complex blocks oestrogen-dependent activation of AMPK

The LKB1–MO25–STRAD complex is capable of targeting the α-catalytic subunit of AMPK at Thr^172^ and activates AMPK by phosphorylation [6–8]. We wished to determine whether oestrogen activation of AMPK is dependent on upstream activation by the LKB1–MO25–STRAD complex. Co-immunoprecipitation with ERα or ERβ in T47D cells revealed the presence of LKB1 as well as MO25 in the elution samples of control and E2-treated cells (Figure 5A). Similar results were found in C2C12 cells and NRCM (Supplementary Figures S4A–S4D). The suggestion is that, at the very least, LKB1 and MO25 of LKB1–MO25–STRAD complex are present in the ER pulldowns and possibly interact with both ERs. To further test the importance of this complex in the activation of AMPK by E2, we silenced MO25 by transfecting T47D cells using siRNA for MO25. First, we confirmed that MO25 mRNA expression level following siRNA treatment was reduced by up to 75% (Supplementary Figure S2D). In Figure 5(B), we showed that genetic deletion of MO25 expression disrupted AMPK phosphorylation by E2 treatment. Moreover, AMPK targeting of ACC was similarly reduced. This shows that E2-dependent activation of AMPK requires the upstream LKB1–MO25–STRAD complex.

**Figure 5:**
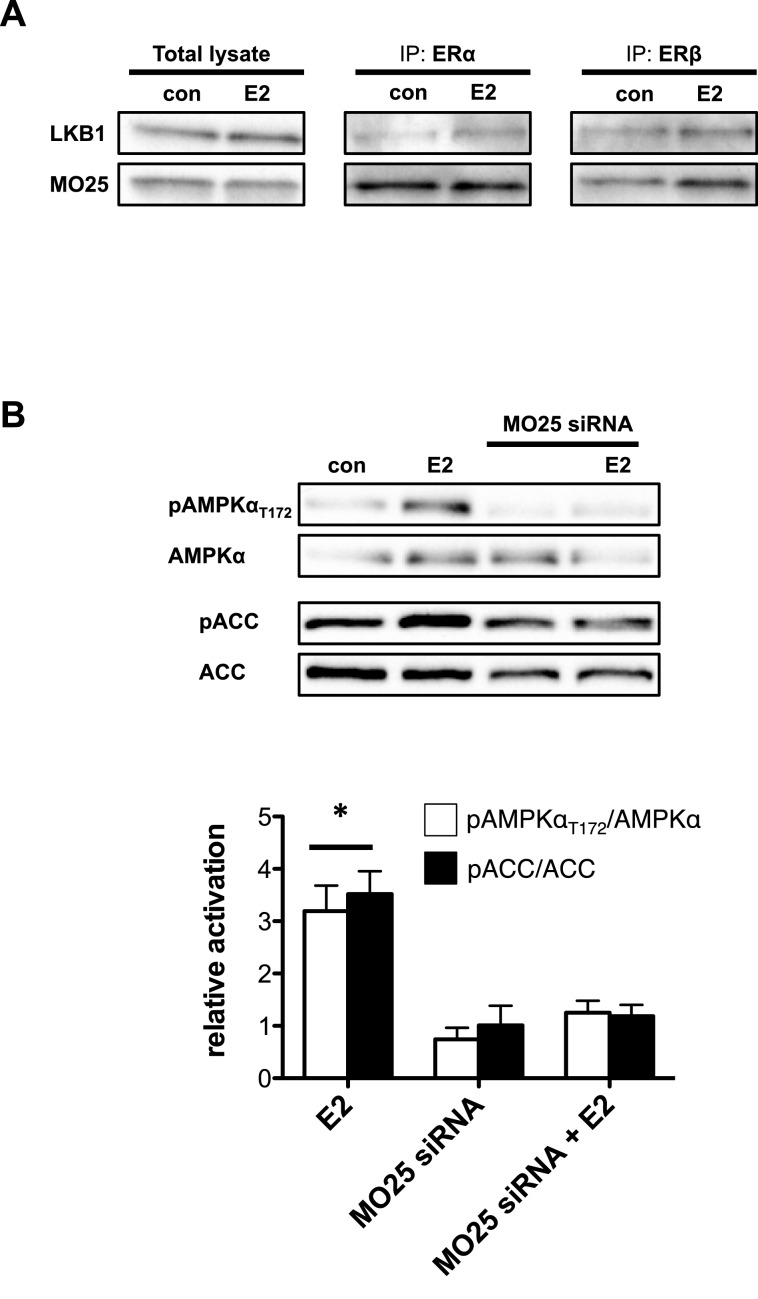
The LKB1 kinase complex is required for E2-dependent activation of AMPK T47D cells (**A**) Anti-ERβ/ERα antibody was used to co-immunoprecipitate (co-IP) endogenous proteins from T47D cells treated with 10 μM E2, followed by western blot analysis for co-IP LKB1 and MO25. (**B**) Western blot (top panel) and bar graph representation (bottom panel) of pAMPKα_T172_–AMPKα ratio and pACC–ACC ratio of total cell lysate from T47D cells transfected with MO25 siRNA. (*P<0.05 from all other groups.)

## DISCUSSION

AMPK is a highly conserved heterotrimeric complex that functions as a versatile metabolic ‘sensor’ with a wide tissue distribution. During pathological conditions, such as cardiac disease, diabetes or cancer, AMPK is central to the metabolic flexibility displayed by the cells in the afflicted organ or tissue. Yet, targeting the AMPK pathway for the development of therapeutics must consider the impact of modifying factors such as sex. This becomes especially critical in pathological settings that display identifiable sex differences such as cardiac disease and diabetes/obesity [40,41]. In addition, breast cancer tumours can be classified as oestrogen-responsive or non-responsive based on the amount and type of ERs found within the resident cancer cells [29]. Moreover, oestrogens are shown to play an important role in the genesis and development of non-small cell lung cancer [42].

Several studies demonstrate that E2 directly and rapidly activates AMPK in multiple cell systems [23–26,43]. The cellular effects of oestrogen are mediated by intracellular ERs (ERα and ERβ). Therefore, we asked the question, which of these two receptors mediate the activation of AMPK. To answer this question, we treated an ERα/β positive human breast cancer cell line (T47D cells) with specific ERα and ERβ agonists. These studies reveal that agonist activation of ERα promotes AMPK activation, measured by an increase in phosphorylation of AMPK and its target ACC, whereas agonist activation of ERβ does not.

Our preliminary data using actinomycin D to inhibit transcriptional activity [44,45] suggest that E2-dependent activation of AMPK is mediated through the non-genomic activity of ERs (Supplementary Figures S5A and S5B). This is not surprising considering the rapid increase in AMPK activation and target phosphorylation and the typical duration for steroid hormones to impart genomic regulation (more than 2 h after stimulation) [46]. Moreover, ∼5%–10% of classical ERs are localized to the plasma membrane, where they activate signal transduction cascades [47,48]. Another membrane receptor that mediates some of the E2-effects at a non-genomic level is a G-protein coupled receptor, GPER (G protein-coupled estrogen receptor 1), previously called GPR30 [22,38,49]. Preliminary data using the GPER antagonist G-15 suggests that AMPK activity does not depend on recruitment of GPER (Supplementary Figures S5C and S5D). Still, a complete time- and dose-dependent series of experiments are required to fully elucidate the contribution of E2–ER genomic and non-genomic, membrane-dependent, pathways to the regulation of AMPK.

We also provide three additional pieces of evidence to verify a specific role for ERα. First, E2 fails to activate AMPK in a cell line that does not express ERα but expresses ERβ (MDA-MB-231). Second, AMPK activation by E2 in T47D cells is not affected by ERβ antagonism; instead, ERβ antagonism potentiates it (to be discussed below). Finally, genetic silencing of ERβ expression in T47D cells does not disrupt E2-dependent activation of AMPK. These results are consistent with previous data indicating that E2 activation of AMPK is mediated through ERα in liver hepatocytes [23]. On the other hand, conflicting results were recently presented demonstrating E2-dependent activation of AMPK that is mediated through ERβ in primary human aortic endothelial cells [43]. Many factors potentially contribute to these discrepancies including the particular cell type, the timing and amount of E2 treatment and the level of resolution (genetic/transcriptional compared with post-translational) for the analysis. Clearly, more studies are required to determine the breadth of E2-dependent activation of the AMPK pathway.

Nevertheless, the seminal finding in the present paper is the interaction of ERs with AMPK. Direct association of the ERs with other proteins such as phosphatidylinositol 3-kinase (PI3K) and p130Cas (Crk-associated substrate) are known to mediate the non-genomic actions of E2 [50–52]. Although AMPK is not considered a scaffold protein, an interactome analysis using a combination of immunoprecipitation and ion trap MS identified 325 proteins that have the potential to interact with AMPKα and 243 for AMPKβ [53]. These proteins are linked to major pathways associated with AMPK, such as metabolism, translation and biosynthesis and are distributed broadly throughout the cytosol, mitochondria, nucleolus and ribosome [53]. Therefore, we hypothesized that E2 regulates the AMPK pathway through direct interaction with AMPK. In a series of co-immunoprecipitation experiments using T47D, C2C12 and NRCM cells, we show that both ERα and ERβ associate with the catalytic α-subunit of AMPK in the presence and absence of E2. We confirm that this is a direct interaction between AMPKα2 and both ERα and ERβ using co-transfection followed by co-immunoprecipitation in 293-T cells that lack functional ERs. The AMPKα2 isoform has previously been shown to attenuate breast cancer progression when overexpressed in tumour cells [54]. It is also the predominant isoform found in skeletal myocytes and cardiomyocytes [55]. Although it shares 77% sequence identity with the AMPKα1 isoform [56], further studies are needed to confirm that AMPKα1 also interacts with the ERs.

Regardless of the isoform type, the α-subunit of AMPK is composed of several domains. Its N-terminal serine/threonine KD (residues 1–312) has the activation loop, which is phosphorylated at Thr^172^ during activation. Next to the KD, there is a putative AID, spanning across residues 313–335 and a C-terminal βγ-subunit-binding domain (residues 396–552) that scaffolds the γ-subunit to the complex [39,57]. A linker sequence (LS) consisting of 60 residues is responsible for connecting the last two domains [58]. Identifying the specific domain where ERs interact with AMPK may provide insight regarding the mechanism by which ERα activates AMPK. Therefore, we co-transfected (293-T cells) specific deletion constructs spanning each of the AMPKα regulatory domains with each ER followed by co-immunoprecipitation with the respective ER. Using these deletion constructs, we were able to tease out the binding domain for both ERα and ERβ within the βγ-subunit-binding domain. This location strategically positions ER binding in close proximity to the regulatory γ-subunit that binds AMP and activates AMPK by eliciting allosteric changes and the β-subunit that may sense the status of cellular energy reserved in the form of glycogen [39,57].

LKB1 is an upstream kinase that phosphorylates the AMPKα subunit, whose activity is dependent on complex formation with two accessory proteins, MO25 and STRAD [59,60]. It also phosphorylates and activates 12 other kinases that are closely related to AMPK [61,62]. A recent study showed that LKB1 associates with ERα and enhances its catalytic transactivation [63]. We found that the LKB1 complex associates with both ERα and ERβ in different cell types, potentially through the scaffolding protein MO25. Moreover, silencing of MO25 suppresses E2-induced activation of AMPK in T47D cells. These findings indicate that transactivation of AMPK by E2 requires the LKB1 kinase complex.

Although the nature of this ER–LKB1 interaction was not determined in the present study, it is known that LKB1 phosphorylation of the α-subunit of AMPK is dependent on allosteric activation of AMPK. Small changes in the AMP–ATP ratio within the cell regulate AMPK activity by modifying the extent of AMP binding to the γ-subunit and leading to conformational changes that enhance the availability of Thr^172^, being permissive to its phosphorylation (or preventing dephosphorylation) [64]. Our finding that LKB1 directly or indirectly associates with ER or ER–AMPK pools suggests that ERs may also modulate LKB1-dependent targeting of AMPK. Co-immunoprecipitation experiments in cells overexpressing components of the LKB1 complex are needed to test whether its association with ERs is a consequence of direct binding.

We did not address whether an alternative AMPK kinase, CAMKKβ, plays a role in T47D cells or other cells investigated in the present study. A recent report identifies CAMKKβ as the main mediator of the E2-dependent activation of AMPK in human endothelial cells [43]. These findings suggest that the mechanism of E2-induced AMPK activation could be cell type-specific and determined by the relative expression of the E2 and AMPK signalling axes components. Further studies are required to delineate the cell specific upstream kinases that meditate E2-dependent activation of AMPK.

Despite a minimal role, if any, for ERβ in E2-mediated activation of AMPK, the fact that ERβ associates with AMPKα similar to ERα implicates a potential regulatory function for ERβ. In our study, inhibition of ERβ potentiates E2 activation of AMPK. We find a similar response in T47D cells where ERβ expression was genetically disrupted by siRNA. This is not surprising considering that a regulatory role for ERβ has been found in other cell systems. In the mammary gland, activation of ERα stimulates cell proliferation that can be antagonized by activation of ERβ [65]. Moreover, proliferation in response to E2 is inhibited in T47D cells stably transfected with ERβ [66]. Although the exact mechanism by which ERβ inhibition potentiates E2 stimulation of AMPK activity is not clear, transient co-expression of ERβ and ERα leads to formation of ER heterodimers [67]. The suggestion is that the βγ-binding domain of AMPKα may act as a nodal point for the integration of ER signalling allowing cells to fine-tune the intensity of E2-dependent AMPK activation by varying the expression level of ERα and ERβ.

Therefore, we propose a model for E2-mediated activation of AMPK (Figure 6). Based on our data, there are at least two possible mechanisms: (A) ERα binds as a heterodimer with ERβ to the α-subunit of AMPK at the βγ-subunit-binding domain and facilitates (**+**) phosphorylation at Thr^172^ by the upstream kinase LKB1; (B) ERα binds as a homodimer with ERα at the same site on AMPKα and more robustly (**+++**) facilitates phosphorylation at Thr^172^ by the upstream kinase LKB1. We show that ER pulldowns contain LKB1 but we do not know whether this is by direct interaction or through association with another protein. Subsequently, downstream targeting by AMPK imitates a changing energetic landscape in the cell.

**Figure 6:**
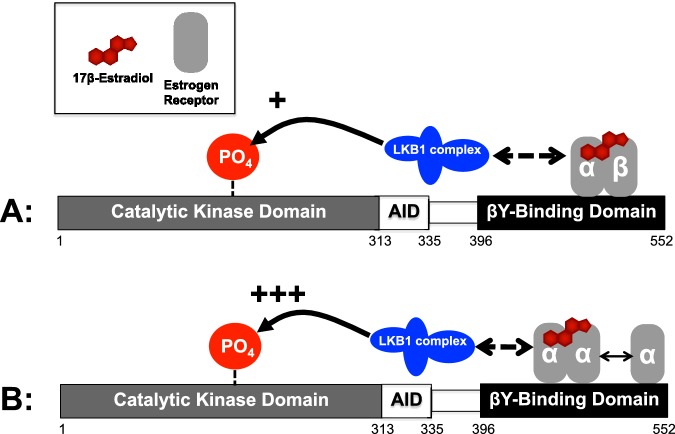
Proposed model for ERs as mediators of AMPK activation AMPKα catalytic subunit binds to ERα or ERβ as a heterodimer (**A**) or a homodimer (**B**) in the presence or absence of E2. Considering that inhibition or genetic deletion of ERβ potentiates E2 activation of AMPK, we predict that the ERs are acting as heterodimers. Next, either through direct or allosteric activation of the LKB1 complex, AMPKα is activated through phosphorylation at Thr^172^ of the catalytic AMPKα subunit.

Our findings do not take away from previous reports that have suggested that E2 metabolites activate AMPK in a different cell system [24]. ER-dependent regulation of AMPK activity could account for only one part of E2-induced AMPK activation. A more complex interplay of direct and indirect effectors modulating AMPK signalling in response to E2 stimulation sure exists. Moreover, ER dependent regulation of AMPK pathway protein expression may also impact on ER responsiveness. Interestingly, ERα has been shown to regulate LKB1 transcription in a cell-context-dependent manner [68,69]. We performed western blot analysis of total cell AMPKα, LKB1 and MO25 contents for the different cell lines used in our study and found similar AMPKα–LKB1 expression ratios between the two cancer cell lines, T47D and MDA-MB-231 (Supplementary Figure S6). However, MDA-MB-231 cells express much higher MO25 levels, when compared with T47D. This variation in MO25 expression could be influenced by differential ER expression in these cell lines and may certainly affect the response of AMPK pathway to external stimuli. Moreover, ER activity could also affect the expression of AMPK subunits α, β and γ.

On the same note, another important question that needs to be addressed is whether E2 signalling alters intracellular AMP pools and, consequently, affects AMPK activity. It becomes especially important, in order to translate these findings into clinical therapeutic targets, the integration of cellular changes in E2-dependent ATP release. Although this is a possibility, our data indicate that any impact of E2-dependent ATP release is integrated with a direct modulation of AMPK signalling by E2. In order to translate these findings into clinical therapeutic targets, a more detailed understanding is required of the site-specific and local regulation of AMPK activity. It would not be surprising if cellular pools of ER–LKB1 or ER–LKB1–AMPK exist. In support of this, western blot data from pull-down experiments using ERα and ERβ coated columns in T47D cells (result not shown) reveal that only total, but not pAMPK associates with the ERs in the absence of E2 stimulation.

In summary, we provide evidence supporting our hypothesis that the mechanism by which E2 activates AMPK is through direct interaction of ERα and ERβ with the α-subunit of AMPK. Preliminary data indicate that ERβ plays a modifying role but the exact mechanism of this function is not known. Our discovery places these two receptors at a nodal point of regulation for cellular energetics. The implication is that many sex dimorphisms in disease, especially in the context of cancer, diabetes and cardiovascular disease, may differentially enroll the AMPK signalling network.

## Abbreviations

ACC: acetyl-CoA carboxylase
AID: auto-inhibitory domain
AMPK: AMP-activated protein kinase
CaMKKβ: calcium–calmodulin-dependent protein kinase kinase β
E2: oestradiol
ER: oestrogen receptor
KD: kinase domain
LKB1: liver kinase B 1
LS: linker sequence
NRCM: neonatal rat cardiomyocytes
TFA: trifluoroacetic acid
